# Knowledge and Clinical Practices of Primary Care Physicians Regarding Soft Tissue Sarcomas: A Descriptive Cross-Sectional Study

**DOI:** 10.3390/healthcare14121700

**Published:** 2026-06-15

**Authors:** Raquel Gracia Rodríguez, Esperanza Romero-Rodríguez, Ignacio Jimena Medina, Fernando Leiva-Cepas

**Affiliations:** 1 Primary Health Care District of Córdoba-Guadalquivir, 14011 Córdoba, Spain; graciarodriguezraquel@gmail.com; 2 Maimonides Institute for Biomedical Research (IMIBIC), 14004 Córdoba, Spain; 3 Department of Morphological and Sociosanitary Sciences, Faculty of Medicine and Nursing, University of Cordoba (UCO), 14071 Córdoba, Spain; cm1jimei@uco.es; 4 Pathology Unit, University Hospital Reina Sofia, 14004 Córdoba, Spain

**Keywords:** sarcoma, primary care, medical education, diagnostic delay, referral pathways, guidelines, survey

## Abstract

**Highlights:**

**What are the main findings?**

- National cross-sectional survey of Primary Care clinicians in Spain (n = 642) identified limited formal undergraduate exposure to softtissue tumors/sarcoma (36% reported none; 17% < 10 h).
- Self-reported knowledge of alarm signs suggestive of sarcoma was low and was higher among younger respondents and medical intern residents than among fully qualified general practitioners.

**What are the implications of the main findings?**

- Targeted primary care education combined with streamlined red-flag referral pathways aligned with national guidelines could facilitate earlier diagnosis of sarcoma.
- Clinicians with prior exposure to oncology training reported greater awareness of soft tissue tumor/sarcoma alarm signs, highlighting the potential impact of targeted educational interventions on early clinical suspicion in routine practice.

**Abstract:**

**Background:** Sarcomas are rare malignant tumors for which diagnostic delay is associated with poorer clinical outcomes. Primary care clinicians (PCCs) are often the first physicians to evaluate patients with suspicious soft tissue masses. This study aimed to assess training exposure, self-reported knowledge of soft tissue tumors/sarcoma (STT/S) alarm signs, and diagnostic practices among PCCs in Spain. **Methods:** We conducted a nationwide descriptive cross-sectional survey between January 2024 and May 2025. A structured questionnaire was distributed through scientific societies, in-person dissemination at healthcare centers, and direct professional contact. Participants included Primary Care physicians (PCPs), and medical intern residents (MIRs). The primary outcome was self-reported knowledge of STT/S alarm signs, assessed as a dichotomous variable. **Results:** A total of 642 clinicians participated, of whom 67% were female. Most respondents were Primary Care general practitioners (64%) or MIRs (31%), and 64% worked in urban settings. Overall, 38% of participants reported being aware of STT/S alarm signs. Undergraduate exposure to oncology- or sarcoma-related content was limited: 36% reported no training, 17% reported fewer than 10 h, and data were missing for 35%. Self-reported knowledge of sarcoma alarm signs was higher among younger participants, residents, and those with prior oncology training. **Conclusions:** Undergraduate exposure to sarcoma-related content and self-reported knowledge of STT/S alarm signs were suboptimal among PCCs in Spain. Targeted educational interventions and simplified referral pathways aligned with national recommendations may help improve earlier recognition and referral of suspected sarcoma.

## 1. Introduction

Soft tissue tumors (STT) comprise a heterogeneous group of mesenchymal neoplasms arising from adipose, muscular, fibrous, vascular, and peripheral nerve tissues. Although the vast majority are benign, their malignant counterparts, soft tissue sarcomas, represent a rare and highly diverse group of tumors accounting for approximately 1% of adult malignancies [1,2].

The diagnosis of sarcoma remains challenging because of its rarity, histological heterogeneity, and frequently non-specific clinical presentation. Delayed diagnosis has consistently been associated with larger tumor size at presentation, more complex treatment strategies, and poorer clinical outcomes [3,4]. In primary care settings, where benign soft tissue lesions are considerably more common than sarcomas, early malignant suspicion may be limited, potentially contributing to delayed investigation and referral [1,5,6,7].

Several referral guidelines and diagnostic pathways have been developed to facilitate earlier recognition of suspicious lesions. Clinical features commonly associated with sarcoma include progressive enlargement, lesion size > 5 cm, deep anatomical location, pain, and recurrence after previous excision [6,8,9]. Tumor depth and rapid growth are considered particularly relevant predictors of malignancy, while persistent unexplained bone pain should raise suspicion for primary bone sarcomas [10]. Current recommendations generally support magnetic resonance imaging (MRI) as the preferred imaging modality for suspicious soft tissue masses and plain radiography as the initial assessment tool for suspected bone lesions, ideally within specialized sarcoma referral pathways to avoid inappropriate biopsy or unplanned excision [2,6].

Primary care physicians (PCPs) are commonly the first clinicians involved in the assessment of patients presenting with soft tissue masses or unexplained musculoskeletal symptoms. Consequently, they play a pivotal role in early suspicion, initial diagnostic orientation, and referral. However, limited exposure to sarcoma during undergraduate and postgraduate medical training, together with the low incidence of these tumors in routine practice, may contribute to diagnostic uncertainty and low familiarity with referral recommendations [3,11,12,13]. Previous studies suggest that focused educational interventions and simplified referral strategies may improve recognition and facilitate earlier specialist assessment [13,14,15].

Despite increasing interest in diagnostic delay and cancer referral pathways, there is still limited evidence specifically addressing sarcoma-related knowledge, training exposure, and self-reported clinical practices among PCPs in Spain [3,4,12,13]. A better understanding of these aspects may help identify educational deficiencies and inform future strategies aimed at improving early recognition and referral of suspected sarcoma in routine clinical practice.

Therefore, the aim of this study was to evaluate knowledge, training exposure, and self-reported diagnostic and referral practices regarding suspected soft tissue sarcoma among primary care physicians in Spain, as well as to explore factors associated with higher knowledge levels.

## 2. Materials and Methods

### 2.1. Study Design

A descriptive, cross-sectional observational study was conducted among PC professionals in Spain.

### 2.2. Setting

The study was carried out at a national level. Data collection took place between January 2024 and May 2025.

### 2.3. Participants

The study population included PCPs and MIRs affiliated with teaching units for medical residency training in Spain, as well as members of the Spanish Society of Primary Care Physicians (SEMERGEN), the Andalusian Society of Family Medicine (SAMFYC), the Spanish Society of Family and Community Medicine (SEMFYC), the Spanish Society of General and Family Physicians (SEMG), and the Spanish Society of Emergency Medicine (SEMES).

Inclusion criteria were being a practicing PCP or MIR during the study period and voluntary agreement to participate. Exclusion criteria included incomplete questionnaires and responses from professionals not currently practicing in PC.

Assuming a 5% alpha error, a precision of 3%, and an expected proportion of 50%, the minimum required sample size was estimated at 1012 participants. A non-probabilistic, voluntary sampling strategy was used.

The voluntary and non-probabilistic nature of the sampling strategy, together with dissemination through scientific societies and professional networks, may have introduced a risk of selection bias, potentially favouring physicians with greater academic or professional engagement.

### 2.4. Questionnaire Development and Validation

Data were collected using a structured questionnaire developed by the research team based on a review of the literature and clinical practice guidelines on soft tissue tumors and bone sarcomas (STT/S).

The instrument comprised four domains:

- Sociodemographic and professional characteristics (e.g., age, sex, years in practice, practice setting, and society membership).
- Training background (e.g., undergraduate exposure to oncology and STT/S, measured in teaching hours).
- Knowledge of STT/S (e.g., familiarity with terminology, recognition of histological subtypes, and identification of alarm signs and symptoms such as rapidly growing soft tissue mass, size > 5 cm, deep location, and persistent enlargement).
- Clinical practice and experience (e.g., prior management of suspected cases, referral patterns, and use of diagnostic tests such as ultrasound, radiography, or CT).

Knowledge and awareness regarding STT/S were explored through multiple questionnaire items covering familiarity with sarcoma-related concepts, previous clinical exposure, diagnostic and referral decisions, awareness of guideline recommendations, and perceptions regarding sarcoma management. Specifically, this assessment included: Q11 (“Please indicate whether you are familiar with the following terms related to soft tissue tumors/sarcomas”; multiple answers allowed); Q13 (“Have you treated patients with this pathology?”); Q14 (“Following clinical suspicion, would you request any diagnostic tests?”; multiple answers allowed); Q15 (“To which specialist would you refer or have you referred these patients?”; multiple answers allowed); Q17 (“Do you participate in tumor boards or multidisciplinary committees in general oncology?”); Q18 (“Are you familiar with the professional attitude and behavior regarding recommendations in the European guidelines?”); Q20 (“Would you consider any of the following measures useful to improve the suspicion and diagnosis of soft tissue tumors/sarcomas?”; multiple answers allowed); and Q21 (“Do you consider the follow-up management of patients with soft tissue tumors/sarcomas in primary care to be appropriate?”).

For questions involving clinical decision-making and management pathways (Q14 and Q15), objective knowledge was assessed using predefined correct responses according to current clinical recommendations. The remaining items reflected self-reported familiarity, previous experience, professional behavior, or attitudes toward sarcoma management. Therefore, knowledge was not measured as a single validated composite score or dichotomized variable but rather explored across multiple complementary domains.

Face and content validity were assessed through consensus among the research team and external experts in primary care and oncology, focusing on clarity, relevance, and comprehensiveness of items. The questionnaire was then pilot-tested in 10 participants with similar characteristics to the target population to evaluate feasibility and comprehension, leading to minor refinements in wording.

### 2.5. Outcome Definitions

The primary outcome was knowledge of STT/S warning signs, operationalized as a dichotomous variable.

A respondent was classified as having adequate knowledge when correctly identifying a predefined set of key alarm features of STT/S, including rapidly growing soft tissue mass, lesion size greater than 5 cm, deep anatomical location, and progressive enlargement or persistence over time.

Secondary variables included self-reported clinical exposure to STT/S cases and referral behavior.

### 2.6. Variables

The study variables included sociodemographic, occupational, training-related, knowledge-related, attitude-related, and practice-related characteristics. Sociodemographic and professional variables comprised age, sex, professional category (attending physician or resident), years of experience in PC, workplace (health center or emergency department), practice setting (rural, semi-urban, or urban), and membership in PC scientific societies (SEMERGEN, SEMG, SAMFYC, SEMFYC, SEMES, or none).

Training-related variables included prior undergraduate education in general oncology and STT/S, measured in teaching hours.

Knowledge-related variables encompassed self-reported knowledge of STT/S, including familiarity with terminology, histological subtypes, and recognition of alarm signs and symptoms.

Practice-related variables included previous experience in the management of patients with STT/S, the number of patients with sarcoma in the physician’s care list, referral patterns to specialist care, and the use of complementary diagnostic tests such as laboratory tests, radiography, ultrasound, or computed tomography. These variables were used to characterize the participants’ real-world clinical management of suspected sarcoma cases.

Attitude-related variables comprised participation in multidisciplinary tumor boards, willingness to be involved in such committees, adherence to European clinical guidelines, and perceptions regarding the usefulness and applicability of these guidelines in clinical decision-making.

The study included two primary outcome variables:

- Knowledge of signs and symptoms suggestive of sarcoma, operationalized as a dichotomous variable (yes/no);
- Clinical practice, assessed through prior experience in the management of patients with sarcoma.

### 2.7. Data Sources and Data Collection

The questionnaire was disseminated through multiple channels, including email distribution via national and regional primary care scientific societies, in-person distribution at primary care centers and residency training units, and through collaboration with Medical Associations across Spain, which facilitated nationwide reach.

Participation was voluntary and anonymous. No reminders were sent. Respondents could optionally provide their name and email address for contact purposes.

Survey responses were automatically recorded using a secure online platform and stored in Google Drive^®^ under restricted access. The data were subsequently exported to Microsoft Excel^®^ for management and statistical analysis. Data confidentiality and traceability were ensured, with access restricted exclusively to the research team.

### 2.8. Statistical Analysis

Both descriptive and inferential statistical analyses were performed for all study variables. Descriptive statistics were used to summarize participant characteristics, and 95% confidence intervals (95% CIs) were calculated for the main study estimators.

Bivariate analyses were conducted to assess associations between independent variables and questionnaire responses. Categorical variables were summarized as absolute frequencies and percentages and compared using Pearson’s chi-square test or Fisher’s exact test, as appropriate. Continuous variables were summarized using means and standard deviations or medians and interquartile ranges, depending on their distributional characteristics and clinical interpretability. When group comparisons involving continuous variables were required, Student’s *t*-test or analysis of variance (ANOVA) was used when the assumptions for parametric analysis were considered reasonable; otherwise, non-parametric alternatives were applied. All statistical tests were two-tailed, and a *p*-value ≤ 0.05 was considered statistically significant.

To identify factors independently associated with higher objective knowledge, a composite knowledge score was constructed based on three questionnaire items (Questions 14, 15, and 18), assessing the initial diagnostic approach to suspected sarcoma, the most appropriate referral specialty, and awareness of European clinical guideline recommendations. One point was assigned for each correct response (score range: 0–3), and participants with a score of ≥2 were classified as having higher knowledge.

Given the clustered nature of the data, with participants nested within Spanish provinces, a preliminary multilevel analysis was performed to assess whether the province variable contributed significantly to the variability of the outcome. As no meaningful between-province effect was identified, a conventional multivariable logistic regression model was fitted.

The dependent variable was higher objective knowledge (yes/no). A fully prespecified multivariable model was used, in which all covariates considered clinically and methodologically relevant were entered simultaneously regardless of their individual statistical significance. These covariates included age, sex, resident status (MIR), membership in a scientific society or teaching unit, and previous university training in oncology and soft tissue tumors. No stepwise or backward elimination procedures were applied.

A complete-case approach was used for the multivariable analysis; only participants with complete information for the outcome and all included covariates were analyzed. Model calibration was assessed using the Hosmer–Leme show goodness-of-fit test.

The minimum required sample size was estimated at 1012 participants, assuming a 5% alpha error, a 50% expected proportion, and a 3% precision level. A total of 642 participants were finally included, resulting in a 37% shortfall relative to the target sample size. Consequently, the predefined level of precision (±3%) was not fully achieved.

Statistical analyses were performed using SPSS 29.2 provided by UCO and MLwiN 3.17 software.

### 2.9. Informed Consent, Ethical and Data Protection Considerations

Participation in the study was voluntary. Before accessing the online questionnaire, participants were provided with an information sheet describing the study objectives, the voluntary nature of participation, and the handling of collected data. Electronic informed consent was obtained through a mandatory checkbox before questionnaire completion. No patient data was collected. Survey responses were stored in an anonymized database with restricted access, available only to the research team. Data were processed in accordance with Regulation (EU) 2016/679 (GDPR) and Spanish Organic Law 3/2018 on personal data protection throughout the study period, from January 2024 to May 2025.

## 3. Results

### 3.1. Sociodemographic and Professional Characteristics

A total of 642 PCPs participated in the study; their main characteristics are summarized in Table 1 and Table 2.

In terms of professional experience, the sample showed a heterogeneous distribution, combining early-career professionals (≤5 years: 33.8%) and highly experienced physicians (>20 years: 42.7%). Most participants worked in urban areas (64%) and in primary healthcare centers (92%), consistent with the study focus.

A high proportion of respondents (76%) reported belonging to at least one scientific society or teaching unit, indicating active engagement in professional development. Regarding training, more than one-third of participants (36%) reported no undergraduate education in oncology or soft tissue tumors, and only a small proportion reported more than 10 h of training, highlighting a relevant gap in formal education.

The sociodemographic, professional, and educational characteristics of the respondents are shown in Table 1. No inferential comparisons were performed in this table, as it was intended to describe the composition of the study sample.

### 3.2. Multivariable Analysis of Factors Associated with Higher Knowledge of Soft Tissue Tumors/Sarcomas

To address the reviewer’s suggestion and to identify independent predictors of higher objective knowledge, we constructed a composite knowledge score based on three key questionnaire items assessing the initial diagnostic approach to suspected sarcoma, the most appropriate referral specialty, and awareness of European clinical guideline recommendations (Questions 14, 15, and 18). Correct responses were defined as follows: (1) requesting radiography and/or computed tomography when sarcoma was suspected; (2) referral to Orthopedic Surgery (locomotor system) and/or Medical Oncology; and (3) self-reported awareness of European clinical guidelines. One point was assigned for each correct response, yielding a total score ranging from 0 to 3. Participants with a score of ≥2 were classified as having higher knowledge, whereas those with scores < 2 were classified as having lower knowledge.

Among the 642 participants included in the study, 227 (35.4%) did not provide valid information regarding prior university training in oncology and soft tissue tumors. This proportion of missing data is reported in Table 1 and should be considered when interpreting analyses involving this variable.

For the multivariable logistic regression analysis, a complete-case approach was used. Only participants with complete information for the composite knowledge outcome and all covariates included in the model were analyzed, resulting in a final sample of 596 participants. No imputation procedures were performed.

Among the 596 respondents included in the complete-case analysis, 581 (97.5%) answered Question 14 correctly, 331 (55.5%) answered Question 15 correctly, and 62 (10.4%) reported awareness of European clinical guideline recommendations. Overall, 355 participants (59.6%) were classified as having higher knowledge.

A multivariable logistic regression model was fitted, including age, sex, resident status (MIR), membership in a scientific society or teaching unit, and previous university training in oncology or soft tissue tumors. The results are presented in Table 3 as adjusted odds ratios (aORs), 95% confidence intervals (95% CIs), and *p*-values. None of the evaluated factors showed statistically significant independent associations with higher knowledge. Male sex showed a non-significant trend toward greater knowledge (aOR 1.38, 95% CI 0.94–2.02; *p* = 0.099). Likewise, age (per 10-year increase: aOR 1.17, 95% CI 0.81–1.68; *p* = 0.411), resident status (aOR 1.27, 95% CI 0.67–2.42; *p* = 0.464), membership in a scientific society or teaching unit (aOR 0.74, 95% CI 0.48–1.12; *p* = 0.153), and previous university training (aOR 1.04, 95% CI 0.72–1.49; *p* = 0.840) were not independently associated with the outcome.

These findings suggest that although several variables appeared to be associated with knowledge in the bivariate analyses, these associations were attenuated after adjustment for potential confounding factors. Given the substantial proportion of missing data in the training variable, the corresponding estimate should be interpreted with caution, as non-random missingness cannot be excluded.

### 3.3. Knowledge of STT/Sarcoma Warning Signs

**Figure 1:**
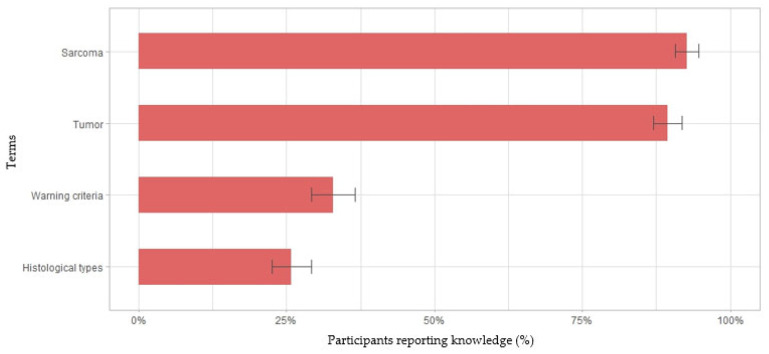
Reported knowledge of key sarcoma-related terms among participating primary care clinicians. Bars represent the proportion of participants reporting knowledge of each term, and error bars indicate 95% confidence intervals for binomial proportions.

Overall, only a minority of participants demonstrated adequate knowledge of the warning signs of sarcomas (Figure 1).

Younger professionals (<30 years) and MIRs demonstrated significantly higher levels of knowledge compared with general practitioners and more experienced physicians (*p* < 0.001). Likewise, professionals with prior university training in oncology or soft tissue tumors were significantly more likely to correctly identify sarcoma warning signs than those without such training (*p* < 0.001).

In addition, belonging to at least one scientific society was associated with higher knowledge levels compared with non-members (*p* < 0.001), suggesting that professional engagement may contribute to improved awareness and recognition of sarcoma-related clinical features.

### 3.4. Clinical Practice and Attitudes Related to Sarcoma Management

Practice- and attitude-related variables revealed variability in the clinical management of patients with suspected sarcoma, as well as in professionals’ perceptions and behaviors (Table 2).

Overall, previous experience in the management of patients with sarcoma was limited, with a substantial proportion of participants reporting no prior exposure. However, some professionals reported occasional experience, particularly in emergency settings. Differences according to sex were observed, with male professionals reporting slightly higher exposure, although the effect size was small (*p* = 0.043; w = 0.103).

Regarding multidisciplinary collaboration, most participants did not currently participate in tumor boards or oncology committees. Nevertheless, a considerable proportion—particularly among female professionals—expressed willingness to be involved, while lack of interest was more frequently reported among male participants (*p* < 0.001; w = 0.125).

No significant differences were observed between sexes in familiarity with European clinical guideline recommendations (*p* > 0.9) or in perceptions of their effectiveness in clinical decision-making (*p* = 0.7). Overall, awareness of these guidelines was limited, although most participants perceived them as useful once considered.

Finally, most professionals supported a shared-care approach for the follow-up of patients with sarcoma, favoring coordination between PC and hospital specialists, with consistent responses across groups (*p* > 0.9).

### 3.5. Statistical and Mathematical Components

The statistical analysis of response distributions was performed using the χ^2^ goodness-of-fit test, which compares the observed frequencies of responses with an expected distribution in which all response categories have equal probability.

## 4. Discussion

This nationwide cross-sectional survey found that knowledge of sarcoma and STT/S warning signs among primary care physicians in Spain was limited overall [1,2,3,4]. In the bivariate analyses, higher levels of knowledge were observed among younger physicians, residents, respondents with prior oncology-related or university training in soft tissue tumors, and those involved in scientific societies. However, these associations were not retained as independent predictors in the multivariable analysis. Undergraduate exposure to STT/S-related content was frequently absent or minimal. Taken together, these findings highlight persistent educational gaps in the early recognition of rare mesenchymal malignancies in frontline clinical settings [14,15,16].

In the multivariable analysis, none of the evaluated factors were independently associated with higher objective knowledge after adjustment. Although younger age, resident status, previous oncology-related training, and membership in scientific societies appeared to be associated with knowledge in the bivariate analyses, these associations were attenuated in the adjusted model. This suggests that the observed subgroup differences should be interpreted cautiously and may reflect overlapping professional, educational, and generational characteristics rather than independent effects of any single variable.

The observed differences in knowledge according to age and training background may reflect variations in exposure to oncology education, updated clinical pathways, and diagnostic recommendations during undergraduate and postgraduate training [17,18]. In contrast, clinicians with longer professional experience may rely more on experiential learning. However, given the rarity and heterogeneity of sarcomas, direct clinical exposure in routine practice remains limited [9,19], which may contribute to lower diagnostic suspicion. These findings are consistent with previous studies reporting higher familiarity with cancer referral pathways among recently trained physicians [17,20,21].

A relevant finding was the limited undergraduate exposure to oncology and STT/S-related content. A substantial proportion of respondents reported no formal training in this area, and only a minority reported more than 10 h of exposure. This suggests an important gap in undergraduate medical education. Similar deficiencies have been reported in both medical students and primary care settings, particularly regarding recognition of soft tissue tumor warning signs and referral criteria [14,20,22]. Given that primary care physicians are often the first point of contact for patients presenting with soft tissue masses or unexplained bone pain, these gaps may be associated with delays in diagnostic suspicion and referral [13,23].

Professional engagement, including membership in scientific societies, was associated with higher levels of awareness of sarcoma warning signs [15,20]. This may reflect differences in access to continuing medical education, updated clinical recommendations, and professional networks. However, given the cross-sectional design and self-reported nature of the data, these findings should be interpreted as associations rather than causal relationships.

From a clinical perspective, limited direct experience in the management of patients with sarcoma was reported, which is expected given the rarity of these tumors [19,24]. In addition, familiarity with European guideline recommendations was suboptimal, although they were generally perceived as useful for clinical decision-making [25,26,27]. These findings suggest that challenges in early sarcoma recognition are not limited to knowledge gaps, but may also relate to the limited dissemination and practical implementation of structured referral pathways in routine care, consistent with current frameworks on early cancer diagnosis and referral processes [15,23]. In this context, concise educational interventions and simplified red-flag referral tools may represent more effective strategies than broad theoretical training alone [13,26].

Overall, awareness of sarcoma warning signs among frontline clinicians in Spain remains suboptimal [13,23]. Strengthening undergraduate exposure, reinforcing continuing medical education, and implementing clear and feasible referral pathways may contribute to improved early suspicion and referral of patients with suspected sarcoma [18,25].

### Limitations

A limitation of this study is the use of a voluntary and non-probabilistic sampling strategy, which may have introduced selection bias and limited the representativeness of the sample. In addition, non-response bias could not be excluded, as the total number of physicians exposed to the survey is unknown.

The study is also based on self-reported knowledge and clinical practice, which may be subject to recall bias and social desirability bias. Furthermore, missing data in some variables, particularly undergraduate oncology training, may have affected the precision of estimates.

Although the study was conducted at a national level, the findings may not be fully generalisable to the broader population of primary care physicians due to the combined effects of voluntary participation and sampling limitations.

Finally, although the questionnaire underwent face and content validation and was pilot-tested, formal psychometric evaluation was not performed. Therefore, the measurement properties of the instrument should be considered when interpreting the findings.

## 5. Conclusions

This nationwide survey suggests that knowledge of STT/S warning signs among frontline clinicians in Spain remains suboptimal. Self-reported awareness was higher among younger physicians, residents, and respondents with prior oncology-related training or involvement in scientific societies, whereas undergraduate exposure to sarcoma-related content was frequently absent or limited. These findings point to persistent educational gaps in the early recognition of STT/S in routine clinical practice.

Given the central role of PCCs in the initial assessment of patients with suspicious soft tissue masses or unexplained bone pain, strengthening undergraduate oncology teaching and promoting targeted continuing medical education may help improve earlier recognition and referral. In addition, the implementation of simple, feasible referral pathways aligned with current recommendations may support more timely specialist evaluation. Future studies should assess whether improving knowledge translates into better diagnostic pathways and reduced delay in sarcoma care.

## Figures and Tables

**Table 1:** Sociodemographic, professional, and educational characteristics of respondents (N = 642).

Characteristic	N = 642 ^1^
Age	
<30	74 (12%)
[30, 44]	219 (34%)
[45, 60]	201 (31%)
>60	127 (20%)
No data	21 (3.0%)
Sex	
Female	428 (67%)
Male	206 (32%)
Others	8 (1.0%)
Profession	
General practitioner in primary care center	413 (64%)
Emergency physician (out-of-hospital)	15 (2.3%)
Emergency physician (hospital-based)	13 (2.0%)
Internal medicine residency	197 (31%)
Others	4 (0.7%)
Years of medical practice	
≤5	217 (33.8%)
(5, 10]	63 (9.8%)
(10, 15]	36 (5.6%)
(15, 20]	50 (7.8%)
>20	274 (42.7%)
No data	2 (0.3%)
Professional practice environment	
Rural (<2000 inhabitants)	86 (13.4%)
Semi-urban (between 2000 and 10,000 inhabitants)	143 (22.3%)
Urban (>10,000 inhabitants)	413 (64.3%)
Workplace	
Hospital emergency services	39 (6.0%)
Out-of-hospital emergency services	13 (2.0%)
Primary healthcare center	590 (92%)
Predominant age range of your patient population	
14–30	9 (1.4%)
31–60	304 (47%)
>60	329 (51%)
Do you belong to any Spanish scientific or teaching unit on PC?	
0	152 (24%)
1	284 (44%)
2 or 3	193 (30%)
4 or more	13 (2.0%)
Total number of patients assigned to your panel	
<1000	50 (7.8%)
1001–1500	197 (31%)
1501–2000	193 (30%)
2001–2500	8 (1.2%)
>2500	7 (1.1%)
Not applicable (emergency physician)	34 (5.3%)
Not applicable (internal medicine residency)	153 (24%)
Have you received any university training in general oncology and softtissue tumors/sarcomas (in hours)?	
0	229 (36%)
<10	106 (17%)
[10, 20)	23 (3.6%)
[20, 50]	41 (6.4%)
>50	16 (2.5%)
No data	227 (35%)
^1^ n (%)	

^1^ n (%); some variables include “No data” when information is missing.

**Table 2:** Clinical experience, guideline awareness, and perceptions regarding management of soft tissue tumors/sarcomas among participating professionals (N = 642).

Characteristic	Female N = 428 ^1^	Male N = 206 ^1^	Others N = 8 ^1^	*p*-Value ^2^	Effect Size
Have you treated patients with this pathology?				0.043	0.103
[1, 3]	120 (28%)	71 (34%)	1 (13%)		
[4, 7]	5 (1.2%)	2 (1.0%)	0 (0%)		
>7	3 (0.7%)	2 (1.0%)	0 (0%)		
No	223 (52%)	78 (38%)	5 (63%)		
Occasionally in the emergency department	77 (18%)	53 (26%)	2 (25%)		
Do you participate in tumor boards or multidisciplinary committees in general oncology?				<0.001	0.125
No, but I believe I should participate	240 (56%)	81 (39%)	7 (88%)		
No, I am not interested in participating	187 (44%)	124 (60%)	1 (13%)		
Yes	1 (0.2%)	1 (0.5%)	0 (0%)		
Are you familiar with the professional’s attitude and behavior regarding therecommendations in the European guidelines?				>0.9	0.037
No	263 (61%)	132 (64%)	6 (75%)		
Unaware of the existence of recommendations	117 (27%)	55 (27%)	2 (25%)		
Yes	48 (11%)	19 (9.2%)	0 (0%)		
Perception of the effectiveness of guidelines and their implications in decision-making				0.7	0.035
Low [0, 3]	6 (1.4%)	4 (1.9%)	0 (0%)		
Medium (3, 7]	141 (33%)	75 (36%)	2 (25%)		
High (7, 10]	281 (66%)	127 (62%)	6 (75%)		
Do you consider the follow-up management of patients with soft tissue tumors/sarcomas in AP to be appropriate?				>0.9	0.028
No, these patients should always be followed by hospital specialists.	33 (7.7%)	16 (7.8%)	0 (0%)		
Others	11 (2.6%)	6 (2.9%)	0 (0%)		
Yes, follow-up of patients with controlled disease should be conducted in coordination with other specialists	384 (90%)	184 (89%)	8 (100%)		

^1^ n (%); median (Q1–Q3) for continuous variables. ^2^ Fisher’s Exact Test for Count Data with simulated *p*-value (based on 1 × 10^5^ replicates). Overall, the sample was predominantly composed of women (67%), with most participants being general practitioners working in PC settings (64%), followed by residents (MIRs) (31%). The age distribution reflected a mainly mid-career workforce, with the majority of participants between 30 and 60 years (65%), while younger professionals (<30 years) and those older than 60 years represented smaller proportions.

**Table 3:** Multivariable Logistic Regression Analysis of Factors Associated with Higher Knowledge of Soft Tissue Tumors/Sarcomas (N = 596).

Variable	Adjusted OR	95% CI	*p*-Value
**Age, per 10-year increase**	1.17	0.81–1.68	0.411
**Male sex**	1.38	0.94–2.02	0.099
**MIR resident status**	1.27	0.67–2.42	0.464
**Membership in scientific society or teaching unit**	0.74	0.48–1.12	0.153
**Previous university training in oncology or soft tissue tumors**	1.04	0.72–1.49	0.840

**Abbreviations:** OR, odds ratio; CI, confidence interval; MIR, medical intern resident. **Model:** dependent variable: higher objective knowledge, defined as composite score ≥2 based on Questions 14, 15, and 18. Complete-case analysis; N = 596.

## Data Availability

The original contributions presented in this study are included in the article. Further inquiries can be directed to the corresponding authors due to privacy and ethical restrictions.

## Abbreviations

The following abbreviations are used in this manuscript:

PCCs	Primary Care Clinicians
STT	Soft Tissue Tumors
PCPs	Primary Care Physicians
PC	Primary Care
NHS	National Health System
MIRs	Medical Internal Residents
STT/S	SoftTissue Tumors/Sarcoma
SEMERGEN	Spanish Society of Primary Care Physicians
SAMFYC	Andalusian Society of Family Medicine
SEMFYC	Spanish Society of Family and Community Medicine
SEMG	Spanish Society of General and Family Physicians
SEMES	Spanish Society of Emergency Medicine
ANOVA	ANalysis Of Variance
SPSS	Statistical Package for Social Sciences
MLwiN	Multilevel Models in Windows
